# Aqua{6,6′-dimeth­oxy-2,2′-[propane-1,3-diylbis(nitrilo­methyl­idyne)]diphenolato}copper(II)

**DOI:** 10.1107/S1600536809042755

**Published:** 2009-10-31

**Authors:** Hui Wang

**Affiliations:** a Department of Chemistry, Mudanjiang Normal College, Mudanjiang 157012, People’s Republic of China

## Abstract

In the asymmetric unit of the title compound, [Cu(C_19_H_20_N_2_O_4_)(H_2_O)], there are two independent mononuclear Cu^II^ complexes. The coordination environment of each Cu^II^ ion is square-pyramidal completed by two N atoms and two O atoms forming the basal plane, and one O atom of the water mol­ecule occupying the apical position. Neighbouring complexes are connected *via* O—H⋯O hydrogen bonds between the water mol­ecule and the meth­oxy group, forming a chain structure along the *a* axis. The propyl­ene groups of the two independent complexes are disordered over two positions with site occupancies of 0.361 (7):0.639 (7) and 0.224 (8):0.776 (8). The crystal under investigation was a partial inversion twin.

## Related literature

For general background to coordination complexes, see: Karlin (1993 ▶); Shankar *et al.* (2009 ▶); Ward (2007 ▶). For a related structure, see: Sui *et al.* (2007 ▶). For the synthesis of the ligand mol­ecule, see: Saha *et al.* (2007 ▶).
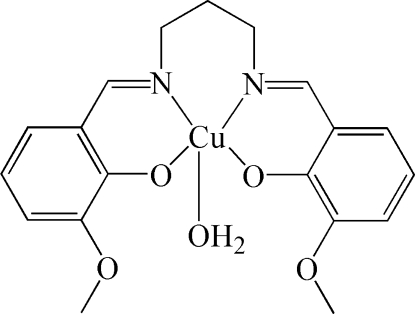


## Experimental

### 

#### Crystal data

[Cu(C_19_H_20_N_2_O_4_)(H_2_O)]
*M* *_r_* = 421.93
Orthorhombic, 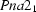

*a* = 20.6870 (15) Å
*b* = 22.9179 (17) Å
*c* = 7.6639 (6) Å
*V* = 3633.5 (5) Å^3^
*Z* = 8
Mo *K*α radiation
μ = 1.24 mm^−1^
*T* = 295 K
0.18 × 0.12 × 0.08 mm

#### Data collection

Bruker APEXII diffractometer
Absorption correction: multi-scan (**SADABS**; Sheldrick, 2003 ▶) *T*_min_ = 0.808, *T*_max_ = 0.908
17668 measured reflections
5909 independent reflections
4814 reflections with *I* > 2σ(*I*)
*R*_int_ = 0.040

#### Refinement

*R*[*F* ^2^ > 2σ(*F* ^2^)] = 0.036
*wR*(*F* ^2^) = 0.085
*S* = 1.00
5909 reflections
502 parameters
15 restraints
H-atom parameters constrained
Δρ_max_ = 0.26 e Å^−3^
Δρ_min_ = −0.29 e Å^−3^
Absolute structure: Flack (1983 ▶), 2453 Friedel pairs
Flack parameter: 0.249 (15)

### 

Data collection: *APEX2* (Bruker, 2004 ▶); cell refinement: *SAINT-Plus* (Bruker, 2001 ▶); data reduction: *SAINT-Plus*; program(s) used to solve structure: *SHELXS97* (Sheldrick, 2008 ▶); program(s) used to refine structure: *SHELXL97* (Sheldrick, 2008 ▶); molecular graphics: *SHELXTL* (Sheldrick, 2008 ▶); software used to prepare material for publication: *SHELXTL*.

## Supplementary Material

Crystal structure: contains datablocks global, I. DOI: 10.1107/S1600536809042755/is2476sup1.cif

Structure factors: contains datablocks I. DOI: 10.1107/S1600536809042755/is2476Isup2.hkl

Additional supplementary materials:  crystallographic information; 3D view; checkCIF report

## Figures and Tables

**Table 1:** Selected bond lengths (Å)

N1—Cu1	1.996 (3)
N2—Cu1	1.988 (4)
N3—Cu2	1.980 (4)
N4—Cu2	1.995 (3)
O2—Cu1	1.958 (3)
O3—Cu1	1.931 (2)
O5—Cu1	2.414 (4)
O7—Cu2	1.934 (3)
O8—Cu2	1.958 (3)
O10—Cu2	2.388 (4)

**Table 2:** Hydrogen-bond geometry (Å, °)

*D*—H⋯*A*	*D*—H	H⋯*A*	*D*⋯*A*	*D*—H⋯*A*
O10—H10*E*⋯O1	0.85	2.00	2.818 (4)	161
O10—H10*F*⋯O4	0.85	2.02	2.768 (4)	147
O5—H5*A*⋯O9^i^	0.85	2.05	2.779 (4)	143
O5—H5*B*⋯O6^i^	0.85	2.02	2.758 (4)	145

Symmetry code: (i) 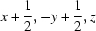.

**Fig. 1.**
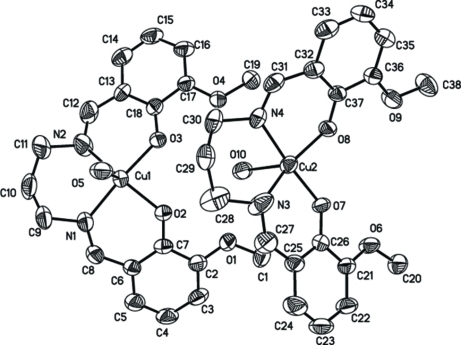
A view of (I) with the atom-labelling scheme. Displacement ellipsoids are drawn at the 50% probability level, and all the hydrogen atoms have been omitted for clarity. In each molecule, only one component of the disordered propylene group is shown.

**Fig. 2.**
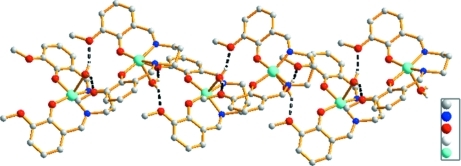
A packing diagram of (I) with a one-dimensional chain-like supramolecular structure, with the hydrogen atoms of carbon atoms being omitted for clarity.

## supplementary crystallographic information

### Comment

Coordination complexes have been intensively researched due to their inherent unique physical and chemical properties (Ward *et al.*, 2007; Shankar *et al.*, 2009). In particular, those compounds with Schiff-base ligands perform at the active sites of many metalloenzymes and therefore play important roles in biological systems (Karlin, 1993). Whereas, it appears necessary to further widen the Schiff-base system of application of metal-organic coordination compounds. Herein, a neutral mononuclear copper(II) compound has been obtained by traditional solution method and its structure is depicted in this paper.

As shown in Fig. 1, compound I is a mononuclear neutral complex [Cu^II^L(H_2_O)] with a distorted molecular configuration. Each Cu(II) ion is coordinated in a square-pyramidal geometry with the basal square built from two nitrogen atoms and two oxygen atoms from *L* ligand, with the apical position occupied by the water molecule. The bond lengths of Cu—O and Cu—N are normal (Sui *et al.*, 2007). The adjacent CuL(H_2_O) molecules are further connected *via* the hydrogen bond O—H···O interaction between the coordinated water molecules and oxygen atoms of alkoxyl group, leading to a one-dimensional chain-like supramolecular structure along the *a* axis in Fig. 2.

### Experimental

The H_2_L ligand was synthesized according to the previous literature (Saha *et al.*, 2007). the synthesis method of the compound I was obtained by allowing the mixture of H_2_L (0.342 g, 1 mmol) and Cu(OAc)_2_.2H_2_O (0.2 g, 1 mmol) being stirred in the 10 ml methanol solution for 30 min, then filtered. The precipitation was collected and dried, the yield of the product is 0.36 g, 85%. Suitable yellow crystals were obtained *via* slow evaporation of the acetone solution containing this complex at room temperature

### Refinement

H atoms were refined using a riding model, with C—H = 0.93–0.97 Å and O—H = 0.85 Å, and with *U*_iso_(H) = 1.2*U*_eq_(C) and 1.5*U*_eq_(methyl C, O). The propylene groups of this crystal were disordered over two positions and the occupancy factors were refined to 0.361 (7):0.639 (7) and 0.224 (8):0.776 (8). The temperature factors of the unprimed and primed atoms of propylene groups have been set to equal by the 'EADP' constraint. The C—C and C—N bond lengths were restrained to 1.52 (1) and 1.50 (1) Å, respectively. The split propylene groups were refined with 'EXYZ' command to share the same C9 atom and C30 atom, respectively. The Flack parameter of 0.249 (15) implies that the crystal used was a partial inversion twin.

### Crystal data

**Table:**

[Cu(C_19_H_20_N_2_O_4_)(H_2_O)]	*F*(000) = 1752
*M**_r_* = 421.93	*D*_x_ = 1.543 Mg m^−^^3^
Orthorhombic, *P**n**a*2_1_	Mo *K*α radiation, λ = 0.71073 Å
Hall symbol: P 2c -2n	Cell parameters from 3860 reflections
*a* = 20.6870 (15) Å	θ = 2.8–24.1°
*b* = 22.9179 (17) Å	µ = 1.24 mm^−^^1^
*c* = 7.6639 (6) Å	*T* = 295 K
*V* = 3633.5 (5) Å^3^	Block, green
*Z* = 8	0.18 × 0.12 × 0.08 mm

### Data collection

**Table:**

Bruker APEXII diffractometer	5909 independent reflections
Radiation source: fine-focus sealed tube	4814 reflections with *I* > 2σ(*I*)
graphite	*R*_int_ = 0.040
Detector resolution: 0 pixels mm^-1^	θ_max_ = 25.0°, θ_min_ = 1.8°
ω scans	*h* = −24→24
Absorption correction: multi-scan (*SADABS*; Sheldrick, 2003)	*k* = −27→25
*T*_min_ = 0.808, *T*_max_ = 0.908	*l* = −9→7
17668 measured reflections	

### Refinement

**Table:**

Refinement on *F*^2^	Secondary atom site location: difference Fourier map
Least-squares matrix: full	Hydrogen site location: inferred from neighbouring sites
*R*[*F*^2^ > 2σ(*F*^2^)] = 0.036	H-atom parameters constrained
*wR*(*F*^2^) = 0.085	*w* = 1/[σ^2^(*F*_o_^2^) + (0.043*P*)^2^] where *P* = (*F*_o_^2^ + 2*F*_c_^2^)/3
*S* = 1.00	(Δ/σ)_max_ < 0.001
5909 reflections	Δρ_max_ = 0.26 e Å^−^^3^
502 parameters	Δρ_min_ = −0.29 e Å^−^^3^
15 restraints	Absolute structure: Flack (1983), 2453 Friedel pairs
Primary atom site location: structure-invariant direct methods	Flack parameter: 0.249 (15)

### Special details

**Table:**

Geometry. All e.s.d.'s (except the e.s.d. in the dihedral angle between two l.s. planes) are estimated using the full covariance matrix. The cell e.s.d.'s are taken into account individually in the estimation of e.s.d.'s in distances, angles and torsion angles; correlations between e.s.d.'s in cell parameters are only used when they are defined by crystal symmetry. An approximate (isotropic) treatment of cell e.s.d.'s is used for estimating e.s.d.'s involving l.s. planes.
Refinement. Refinement of *F*^2^ against ALL reflections. The weighted *R*-factor *wR* and goodness of fit *S* are based on *F*^2^, conventional *R*-factors *R* are based on *F*, with *F* set to zero for negative *F*^2^. The threshold expression of *F*^2^ > σ(*F*^2^) is used only for calculating *R*-factors(gt) *etc*. and is not relevant to the choice of reflections for refinement. *R*-factors based on *F*^2^ are statistically about twice as large as those based on *F*, and *R*- factors based on ALL data will be even larger.

### Fractional atomic coordinates and isotropic or equivalent isotropic displacement parameters (Å^2^)

**Table:**

	*x*	*y*	*z*	*U*_iso_*/*U*_eq_	Occ. (<1)
C1	0.09091 (19)	0.3864 (2)	0.1707 (7)	0.0648 (14)	
H1A	0.0616	0.3568	0.2113	0.097*	
H1B	0.0794	0.3976	0.0541	0.097*	
H1C	0.0884	0.4197	0.2461	0.097*	
C2	0.20370 (19)	0.39932 (16)	0.1185 (6)	0.0432 (10)	
C3	0.1964 (2)	0.45593 (17)	0.0630 (7)	0.0537 (12)	
H3	0.1552	0.4723	0.0612	0.064*	
C4	0.2486 (2)	0.48950 (19)	0.0095 (6)	0.0609 (13)	
H4	0.2426	0.5277	−0.0283	0.073*	
C5	0.3084 (2)	0.46558 (19)	0.0136 (6)	0.0575 (14)	
H5	0.3438	0.4881	−0.0192	0.069*	
C6	0.31810 (19)	0.40757 (16)	0.0661 (6)	0.0414 (10)	
C7	0.26648 (18)	0.37226 (15)	0.1208 (6)	0.0383 (10)	
C8	0.38423 (18)	0.38649 (18)	0.0719 (6)	0.0446 (10)	
H8	0.4163	0.4143	0.0538	0.054*	
C9	0.47463 (17)	0.32791 (18)	0.1093 (6)	0.0542 (12)	0.361 (7)
H9A	0.4868	0.3166	0.2267	0.065*	0.361 (7)
H9B	0.4954	0.3648	0.0830	0.065*	0.361 (7)
C10	0.4971 (11)	0.2816 (5)	−0.020 (2)	0.070 (3)	0.361 (7)
H10A	0.4796	0.2910	−0.1341	0.085*	0.361 (7)
H10B	0.5439	0.2835	−0.0288	0.085*	0.361 (7)
C11	0.4782 (3)	0.2195 (6)	0.024 (2)	0.074 (3)	0.361 (7)
H11A	0.4884	0.2110	0.1453	0.089*	0.361 (7)
H11B	0.5014	0.1922	−0.0493	0.089*	0.361 (7)
C9'	0.47463 (17)	0.32791 (18)	0.1093 (6)	0.0542 (12)	0.639 (7)
H9C	0.4881	0.3369	0.2275	0.065*	0.639 (7)
H9D	0.4944	0.3566	0.0329	0.065*	0.639 (7)
C10'	0.5005 (6)	0.2684 (4)	0.0615 (14)	0.070 (3)	0.639 (7)
H10C	0.4962	0.2420	0.1598	0.085*	0.639 (7)
H10D	0.5460	0.2712	0.0313	0.085*	0.639 (7)
C11'	0.4622 (4)	0.2455 (4)	−0.0931 (12)	0.074 (3)	0.639 (7)
H11C	0.4877	0.2186	−0.1621	0.089*	0.639 (7)
H11D	0.4476	0.2772	−0.1673	0.089*	0.639 (7)
C12	0.3935 (2)	0.1614 (2)	−0.0492 (7)	0.0614 (14)	
H12	0.4262	0.1423	−0.1094	0.074*	
C13	0.33653 (18)	0.12826 (18)	−0.0148 (6)	0.0397 (10)	
C14	0.3349 (2)	0.0695 (2)	−0.0731 (6)	0.0558 (13)	
H14	0.3701	0.0546	−0.1340	0.067*	
C15	0.2833 (2)	0.03444 (19)	−0.0423 (7)	0.0598 (13)	
H15	0.2829	−0.0038	−0.0829	0.072*	
C16	0.2310 (2)	0.05641 (17)	0.0507 (6)	0.0540 (13)	
H16	0.1957	0.0324	0.0734	0.065*	
C17	0.23071 (17)	0.11327 (15)	0.1100 (6)	0.0397 (10)	
C18	0.28444 (17)	0.15138 (16)	0.0825 (6)	0.0380 (10)	
C19	0.12368 (18)	0.10614 (19)	0.2255 (7)	0.0524 (12)	
H19A	0.0934	0.1291	0.2910	0.079*	
H19B	0.1335	0.0711	0.2889	0.079*	
H19C	0.1051	0.0962	0.1146	0.079*	
C20	−0.12773 (19)	0.39180 (19)	0.4322 (6)	0.0532 (12)	
H20A	−0.1594	0.3660	0.3832	0.080*	
H20B	−0.1205	0.4238	0.3538	0.080*	
H20C	−0.1430	0.4064	0.5421	0.080*	
C21	−0.01810 (18)	0.39057 (17)	0.5282 (6)	0.0412 (11)	
C22	−0.01621 (19)	0.44966 (16)	0.5539 (6)	0.0519 (12)	
H22	−0.0518	0.4724	0.5245	0.062*	
C23	0.0383 (2)	0.47585 (17)	0.6234 (7)	0.0594 (13)	
H23	0.0397	0.5161	0.6393	0.071*	
C24	0.0896 (2)	0.44249 (19)	0.6679 (7)	0.0598 (13)	
H24	0.1260	0.4603	0.7148	0.072*	
C25	0.08947 (18)	0.38171 (16)	0.6454 (6)	0.0417 (11)	
C26	0.03530 (18)	0.35374 (16)	0.5714 (6)	0.0370 (9)	
C27	0.1449 (2)	0.3496 (2)	0.7007 (7)	0.0588 (14)	
H27	0.1785	0.3715	0.7479	0.071*	
C28	0.2259 (4)	0.2890 (9)	0.661 (3)	0.065 (2)	0.224 (8)
H28A	0.2350	0.2926	0.5375	0.078*	0.224 (8)
H28B	0.2491	0.3196	0.7224	0.078*	0.224 (8)
C29	0.2480 (18)	0.2297 (6)	0.726 (3)	0.065 (3)	0.224 (8)
H29A	0.2319	0.2243	0.8440	0.078*	0.224 (8)
H29B	0.2948	0.2295	0.7317	0.078*	0.224 (8)
C30	0.22592 (17)	0.17823 (18)	0.6147 (7)	0.0533 (11)	0.224 (8)
H30A	0.2378	0.1848	0.4939	0.064*	0.224 (8)
H30B	0.2470	0.1428	0.6541	0.064*	0.224 (8)
C28'	0.2125 (3)	0.2698 (3)	0.7848 (9)	0.065 (2)	0.776 (8)
H28C	0.2382	0.3005	0.8370	0.078*	0.776 (8)
H28D	0.1995	0.2426	0.8753	0.078*	0.776 (8)
C29'	0.2498 (4)	0.2390 (3)	0.6439 (12)	0.065 (3)	0.776 (8)
H29C	0.2462	0.2609	0.5361	0.078*	0.776 (8)
H29D	0.2952	0.2376	0.6761	0.078*	0.776 (8)
C30'	0.22592 (17)	0.17823 (18)	0.6147 (7)	0.0533 (11)	0.776 (8)
H30C	0.2392	0.1656	0.4994	0.064*	0.776 (8)
H30D	0.2463	0.1526	0.6990	0.064*	0.776 (8)
C31	0.1352 (2)	0.12165 (19)	0.6818 (6)	0.0440 (11)	
H31	0.1672	0.0960	0.7194	0.053*	
C32	0.0696 (2)	0.10016 (17)	0.6892 (6)	0.0404 (10)	
C33	0.0592 (2)	0.04488 (18)	0.7611 (6)	0.0517 (12)	
H33	0.0941	0.0247	0.8083	0.062*	
C34	−0.0003 (2)	0.01979 (19)	0.7640 (6)	0.0554 (12)	
H34	−0.0065	−0.0165	0.8159	0.067*	
C35	−0.0517 (2)	0.04940 (17)	0.6881 (6)	0.0464 (11)	
H35	−0.0923	0.0320	0.6858	0.056*	
C36	−0.04381 (18)	0.10348 (16)	0.6167 (6)	0.0406 (10)	
C37	0.01811 (17)	0.13165 (15)	0.6142 (5)	0.0340 (9)	
C38	−0.1553 (2)	0.1113 (2)	0.5384 (8)	0.0673 (15)	
H38A	−0.1840	0.1379	0.4803	0.101*	
H38B	−0.1694	0.1057	0.6565	0.101*	
H38C	−0.1553	0.0746	0.4785	0.101*	
N1	0.40348 (14)	0.33463 (14)	0.0987 (6)	0.0426 (8)	
N2	0.40470 (19)	0.2144 (2)	−0.0068 (6)	0.0720 (14)	
N3	0.15392 (17)	0.29488 (18)	0.6935 (6)	0.0686 (14)	
N4	0.15466 (14)	0.17189 (14)	0.6296 (6)	0.0396 (8)	
O1	0.15456 (12)	0.36417 (12)	0.1717 (5)	0.0558 (9)	
O2	0.27165 (12)	0.31825 (11)	0.1707 (4)	0.0422 (8)	
O3	0.28187 (12)	0.20415 (10)	0.1451 (4)	0.0390 (7)	
O4	0.18053 (13)	0.13826 (12)	0.1993 (5)	0.0529 (8)	
O5	0.39293 (18)	0.25291 (11)	0.4074 (6)	0.0525 (11)	
H5A	0.4151	0.2810	0.4484	0.079*	
H5B	0.4174	0.2234	0.3959	0.079*	
O6	−0.06950 (13)	0.36132 (12)	0.4577 (5)	0.0582 (10)	
O7	0.03113 (12)	0.29827 (11)	0.5386 (4)	0.0415 (8)	
O8	0.02381 (12)	0.18312 (11)	0.5450 (4)	0.0402 (8)	
O9	−0.09157 (13)	0.13489 (12)	0.5383 (5)	0.0558 (10)	
O10	0.14401 (17)	0.24940 (12)	0.3006 (6)	0.0520 (10)	
H10E	0.1567	0.2828	0.2672	0.078*	
H10F	0.1663	0.2239	0.2471	0.078*	
Cu1	0.34458 (2)	0.26616 (2)	0.12353 (6)	0.03785 (14)	
Cu2	0.09494 (2)	0.238236 (18)	0.58068 (6)	0.03693 (15)	

### Atomic displacement parameters (Å^2^)

**Table:**

	*U*^11^	*U*^22^	*U*^33^	*U*^12^	*U*^13^	*U*^23^
C1	0.045 (3)	0.071 (3)	0.078 (4)	0.018 (2)	0.009 (3)	0.013 (3)
C2	0.051 (2)	0.037 (2)	0.042 (3)	0.0014 (18)	0.006 (2)	0.000 (2)
C3	0.060 (3)	0.039 (2)	0.062 (3)	0.017 (2)	0.004 (3)	0.002 (2)
C4	0.078 (3)	0.030 (2)	0.074 (4)	0.006 (2)	0.014 (3)	0.014 (2)
C5	0.064 (3)	0.041 (3)	0.067 (4)	−0.002 (2)	0.015 (3)	0.006 (2)
C6	0.045 (2)	0.033 (2)	0.046 (3)	−0.0015 (17)	0.004 (2)	0.005 (2)
C7	0.048 (2)	0.030 (2)	0.038 (2)	0.0019 (16)	−0.006 (2)	0.0008 (19)
C8	0.043 (2)	0.044 (2)	0.047 (3)	−0.0075 (18)	0.001 (2)	0.006 (2)
C9	0.033 (2)	0.068 (3)	0.062 (3)	−0.0062 (19)	−0.004 (2)	0.011 (3)
C10	0.030 (3)	0.090 (6)	0.092 (10)	0.006 (4)	−0.007 (7)	0.010 (6)
C11	0.054 (5)	0.073 (6)	0.096 (9)	−0.011 (4)	0.053 (6)	−0.005 (4)
C9'	0.033 (2)	0.068 (3)	0.062 (3)	−0.0062 (19)	−0.004 (2)	0.011 (3)
C10'	0.030 (3)	0.090 (6)	0.092 (10)	0.006 (4)	−0.007 (7)	0.010 (6)
C11'	0.054 (5)	0.073 (6)	0.096 (9)	−0.011 (4)	0.053 (6)	−0.005 (4)
C12	0.051 (3)	0.072 (4)	0.061 (3)	0.005 (3)	0.018 (3)	−0.020 (3)
C13	0.039 (2)	0.044 (3)	0.036 (2)	0.0128 (19)	0.0032 (19)	−0.0019 (19)
C14	0.064 (3)	0.049 (3)	0.055 (3)	0.017 (2)	0.009 (2)	−0.010 (2)
C15	0.075 (4)	0.033 (3)	0.072 (3)	0.009 (2)	0.003 (3)	−0.017 (2)
C16	0.059 (3)	0.032 (2)	0.071 (4)	0.0000 (19)	−0.002 (3)	−0.009 (2)
C17	0.041 (2)	0.033 (2)	0.045 (3)	0.0018 (16)	−0.001 (2)	−0.004 (2)
C18	0.037 (2)	0.039 (2)	0.038 (3)	0.0045 (16)	−0.003 (2)	−0.001 (2)
C19	0.041 (3)	0.046 (3)	0.069 (3)	−0.007 (2)	−0.001 (2)	−0.007 (2)
C20	0.038 (2)	0.053 (3)	0.069 (3)	0.007 (2)	−0.012 (2)	−0.001 (2)
C21	0.038 (2)	0.034 (2)	0.051 (3)	−0.0003 (17)	−0.001 (2)	−0.0074 (18)
C22	0.050 (2)	0.032 (2)	0.074 (3)	0.0069 (18)	−0.010 (3)	−0.005 (2)
C23	0.067 (3)	0.032 (2)	0.079 (4)	−0.0019 (19)	−0.009 (3)	−0.013 (2)
C24	0.056 (3)	0.047 (3)	0.077 (4)	−0.008 (2)	−0.019 (3)	−0.016 (2)
C25	0.041 (2)	0.036 (2)	0.047 (3)	−0.0028 (17)	−0.006 (2)	−0.0094 (18)
C26	0.037 (2)	0.033 (2)	0.041 (2)	−0.0019 (16)	0.003 (2)	−0.001 (2)
C27	0.045 (3)	0.056 (3)	0.075 (4)	−0.002 (2)	−0.022 (3)	−0.026 (3)
C28	0.077 (5)	0.067 (4)	0.051 (5)	0.020 (3)	−0.046 (4)	−0.013 (4)
C29	0.028 (3)	0.070 (4)	0.098 (8)	0.002 (3)	−0.008 (6)	0.002 (5)
C30	0.031 (2)	0.067 (3)	0.061 (3)	0.0114 (19)	−0.005 (2)	0.002 (3)
C28'	0.077 (5)	0.067 (4)	0.051 (5)	0.020 (3)	−0.046 (4)	−0.013 (4)
C29'	0.028 (3)	0.070 (4)	0.098 (8)	0.002 (3)	−0.008 (6)	0.002 (5)
C30'	0.031 (2)	0.067 (3)	0.061 (3)	0.0114 (19)	−0.005 (2)	0.002 (3)
C31	0.047 (2)	0.044 (3)	0.041 (3)	0.016 (2)	−0.003 (2)	0.005 (2)
C32	0.046 (2)	0.037 (2)	0.039 (2)	0.0059 (19)	−0.0042 (19)	0.0051 (18)
C33	0.059 (3)	0.045 (3)	0.051 (3)	0.016 (2)	−0.007 (2)	0.006 (2)
C34	0.068 (3)	0.033 (3)	0.065 (3)	−0.002 (2)	0.003 (3)	0.014 (2)
C35	0.051 (3)	0.040 (2)	0.048 (3)	−0.007 (2)	0.001 (2)	0.0029 (19)
C36	0.044 (2)	0.035 (2)	0.043 (3)	−0.0004 (17)	0.002 (2)	0.003 (2)
C37	0.039 (2)	0.030 (2)	0.033 (2)	0.0027 (15)	−0.002 (2)	0.0076 (19)
C38	0.047 (3)	0.069 (3)	0.085 (4)	−0.014 (2)	−0.014 (3)	0.025 (3)
N1	0.0345 (17)	0.049 (2)	0.044 (2)	−0.0029 (14)	−0.0020 (17)	0.008 (2)
N2	0.062 (3)	0.068 (3)	0.086 (4)	−0.015 (2)	0.044 (2)	−0.025 (3)
N3	0.050 (2)	0.058 (3)	0.098 (4)	0.0197 (19)	−0.032 (2)	−0.033 (2)
N4	0.0313 (17)	0.047 (2)	0.040 (2)	0.0097 (14)	−0.0013 (16)	0.001 (2)
O1	0.0397 (17)	0.0407 (17)	0.087 (3)	0.0081 (13)	0.0083 (17)	0.0134 (16)
O2	0.0392 (16)	0.0323 (15)	0.055 (2)	0.0026 (12)	0.0037 (14)	0.0065 (13)
O3	0.0389 (15)	0.0324 (14)	0.0457 (18)	−0.0009 (11)	0.0060 (14)	−0.0097 (13)
O4	0.0419 (17)	0.0345 (16)	0.082 (2)	−0.0051 (13)	0.0141 (17)	−0.0170 (15)
O5	0.059 (2)	0.0342 (17)	0.065 (3)	−0.0006 (13)	−0.012 (2)	−0.0024 (14)
O6	0.0328 (16)	0.0343 (16)	0.107 (3)	0.0049 (13)	−0.0192 (18)	−0.0152 (16)
O7	0.0323 (14)	0.0268 (15)	0.065 (2)	0.0035 (11)	−0.0069 (14)	−0.0099 (13)
O8	0.0331 (14)	0.0320 (15)	0.055 (2)	0.0011 (11)	−0.0059 (14)	0.0099 (13)
O9	0.0394 (17)	0.0471 (17)	0.081 (3)	−0.0063 (14)	−0.0120 (17)	0.0226 (16)
O10	0.061 (2)	0.0386 (19)	0.056 (3)	0.0058 (14)	0.017 (2)	0.0035 (13)
Cu1	0.0328 (3)	0.0381 (3)	0.0426 (4)	−0.0014 (2)	0.0035 (2)	0.0004 (3)
Cu2	0.0321 (3)	0.0362 (2)	0.0425 (4)	0.0051 (2)	−0.0054 (2)	−0.0022 (3)

### Geometric parameters (Å, °)

**Table:**

C1—O1	1.412 (4)	C22—C23	1.385 (5)
C1—H1A	0.9600	C22—H22	0.9300
C1—H1B	0.9600	C23—C24	1.351 (6)
C1—H1C	0.9600	C23—H23	0.9300
C2—O1	1.360 (4)	C24—C25	1.404 (5)
C2—C3	1.374 (5)	C24—H24	0.9300
C2—C7	1.439 (5)	C25—C26	1.410 (5)
C3—C4	1.388 (6)	C25—C27	1.426 (6)
C3—H3	0.9300	C26—O7	1.299 (4)
C4—C5	1.353 (6)	C27—N3	1.270 (6)
C4—H4	0.9300	C27—H27	0.9300
C5—C6	1.404 (5)	C28—C29	1.516 (10)
C5—H5	0.9300	C28—N3	1.516 (6)
C6—C7	1.404 (5)	C28—H28A	0.9700
C6—C8	1.452 (5)	C28—H28B	0.9700
C7—O2	1.300 (4)	C29—C30	1.527 (10)
C8—N1	1.270 (5)	C29—H29A	0.9700
C8—H8	0.9300	C29—H29B	0.9700
C9—N1	1.482 (4)	C30—N4	1.486 (4)
C9—C10	1.526 (10)	C30—H30A	0.9700
C9—H9A	0.9700	C30—H30B	0.9700
C9—H9B	0.9700	C28'—C29'	1.504 (7)
C10—C11	1.513 (10)	C28'—N3	1.513 (5)
C10—H10A	0.9700	C28'—H28C	0.9700
C10—H10B	0.9700	C28'—H28D	0.9700
C11—N2	1.543 (6)	C29'—H29C	0.9700
C11—H11A	0.9700	C29'—H29D	0.9700
C11—H11B	0.9700	C31—N4	1.284 (5)
C10'—C11'	1.520 (8)	C31—C32	1.445 (6)
C10'—H10C	0.9700	C31—H31	0.9300
C10'—H10D	0.9700	C32—C33	1.398 (5)
C11'—N2	1.536 (5)	C32—C37	1.409 (5)
C11'—H11C	0.9700	C33—C34	1.359 (6)
C11'—H11D	0.9700	C33—H33	0.9300
C12—N2	1.277 (6)	C34—C35	1.389 (6)
C12—C13	1.427 (6)	C34—H34	0.9300
C12—H12	0.9300	C35—C36	1.364 (5)
C13—C18	1.413 (5)	C35—H35	0.9300
C13—C14	1.420 (6)	C36—O9	1.362 (5)
C14—C15	1.357 (6)	C36—C37	1.435 (5)
C14—H14	0.9300	C37—O8	1.299 (4)
C15—C16	1.390 (6)	C38—O9	1.424 (4)
C15—H15	0.9300	C38—H38A	0.9600
C16—C17	1.380 (5)	C38—H38B	0.9600
C16—H16	0.9300	C38—H38C	0.9600
C17—O4	1.369 (5)	N1—Cu1	1.996 (3)
C17—C18	1.429 (5)	N2—Cu1	1.988 (4)
C18—O3	1.302 (4)	N3—Cu2	1.980 (4)
C19—O4	1.402 (4)	N4—Cu2	1.995 (3)
C19—H19A	0.9600	O2—Cu1	1.958 (3)
C19—H19B	0.9600	O3—Cu1	1.931 (2)
C19—H19C	0.9600	O5—Cu1	2.414 (4)
C20—O6	1.406 (5)	O5—H5A	0.8499
C20—H20A	0.9600	O5—H5B	0.8500
C20—H20B	0.9600	O7—Cu2	1.934 (3)
C20—H20C	0.9600	O8—Cu2	1.958 (3)
C21—O6	1.368 (5)	O10—Cu2	2.388 (4)
C21—C22	1.369 (5)	O10—H10E	0.8500
C21—C26	1.429 (5)	O10—H10F	0.8499
			
O1—C1—H1A	109.5	N3—C27—H27	116.0
O1—C1—H1B	109.5	C25—C27—H27	116.0
H1A—C1—H1B	109.5	C29—C28—N3	108.8 (19)
O1—C1—H1C	109.5	C29—C28—H28A	109.9
H1A—C1—H1C	109.5	N3—C28—H28A	109.9
H1B—C1—H1C	109.5	C29—C28—H28B	109.9
O1—C2—C3	124.8 (4)	N3—C28—H28B	109.9
O1—C2—C7	114.6 (3)	H28A—C28—H28B	108.3
C3—C2—C7	120.7 (4)	C28—C29—C30	114.7 (15)
C2—C3—C4	122.0 (4)	C28—C29—H29A	108.6
C2—C3—H3	119.0	C30—C29—H29A	108.6
C4—C3—H3	119.0	C28—C29—H29B	108.6
C5—C4—C3	118.7 (4)	C30—C29—H29B	108.6
C5—C4—H4	120.7	H29A—C29—H29B	107.6
C3—C4—H4	120.7	N4—C30—C29	109.2 (15)
C4—C5—C6	121.4 (4)	N4—C30—H30A	109.8
C4—C5—H5	119.3	C29—C30—H30A	109.8
C6—C5—H5	119.3	N4—C30—H30B	109.8
C7—C6—C5	121.5 (4)	C29—C30—H30B	109.8
C7—C6—C8	121.1 (3)	H30A—C30—H30B	108.3
C5—C6—C8	117.3 (4)	C29'—C28'—N3	105.0 (6)
O2—C7—C6	125.0 (3)	C29'—C28'—H28C	110.8
O2—C7—C2	119.2 (3)	N3—C28'—H28C	110.8
C6—C7—C2	115.8 (3)	C29'—C28'—H28D	110.8
N1—C8—C6	127.7 (4)	N3—C28'—H28D	110.8
N1—C8—H8	116.1	H28C—C28'—H28D	108.8
C6—C8—H8	116.1	C28'—C29'—H29C	109.2
N1—C9—C10	109.9 (10)	C28'—C29'—H29D	109.2
N1—C9—H9A	109.7	H29C—C29'—H29D	107.9
C10—C9—H9A	109.7	N4—C31—C32	127.7 (4)
N1—C9—H9B	109.7	N4—C31—H31	116.1
C10—C9—H9B	109.7	C32—C31—H31	116.1
H9A—C9—H9B	108.2	C33—C32—C37	120.6 (4)
C11—C10—C9	115.4 (12)	C33—C32—C31	117.9 (4)
C11—C10—H10A	108.4	C37—C32—C31	121.3 (4)
C9—C10—H10A	108.4	C34—C33—C32	121.9 (4)
C11—C10—H10B	108.4	C34—C33—H33	119.0
C9—C10—H10B	108.4	C32—C33—H33	119.0
H10A—C10—H10B	107.5	C33—C34—C35	118.7 (4)
C10—C11—N2	107.0 (13)	C33—C34—H34	120.7
C10—C11—H11A	110.3	C35—C34—H34	120.7
N2—C11—H11A	110.3	C36—C35—C34	121.4 (4)
C10—C11—H11B	110.3	C36—C35—H35	119.3
N2—C11—H11B	110.3	C34—C35—H35	119.3
H11A—C11—H11B	108.6	O9—C36—C35	124.8 (3)
C11'—C10'—H10C	110.0	O9—C36—C37	113.8 (3)
C11'—C10'—H10D	110.0	C35—C36—C37	121.4 (4)
H10C—C10'—H10D	108.4	O8—C37—C32	124.3 (3)
C10'—C11'—N2	103.2 (8)	O8—C37—C36	119.7 (3)
C10'—C11'—H11C	111.1	C32—C37—C36	116.0 (3)
N2—C11'—H11C	111.1	O9—C38—H38A	109.5
C10'—C11'—H11D	111.1	O9—C38—H38B	109.5
N2—C11'—H11D	111.1	H38A—C38—H38B	109.5
H11C—C11'—H11D	109.1	O9—C38—H38C	109.5
N2—C12—C13	127.5 (4)	H38A—C38—H38C	109.5
N2—C12—H12	116.2	H38B—C38—H38C	109.5
C13—C12—H12	116.2	C8—N1—C9	114.7 (3)
C18—C13—C14	120.3 (4)	C8—N1—Cu1	124.0 (3)
C18—C13—C12	121.8 (4)	C9—N1—Cu1	121.3 (3)
C14—C13—C12	117.8 (4)	C12—N2—C11'	118.2 (5)
C15—C14—C13	121.7 (4)	C12—N2—C11	106.9 (7)
C15—C14—H14	119.2	C12—N2—Cu1	125.5 (3)
C13—C14—H14	119.2	C11'—N2—Cu1	115.0 (4)
C14—C15—C16	119.2 (4)	C11—N2—Cu1	119.5 (7)
C14—C15—H15	120.4	C27—N3—C28'	118.3 (4)
C16—C15—H15	120.4	C27—N3—C28	103.9 (8)
C17—C16—C15	121.0 (4)	C27—N3—Cu2	125.2 (3)
C17—C16—H16	119.5	C28'—N3—Cu2	116.5 (3)
C15—C16—H16	119.5	C28—N3—Cu2	118.4 (8)
O4—C17—C16	124.3 (3)	C31—N4—C30	115.0 (3)
O4—C17—C18	114.1 (3)	C31—N4—Cu2	123.3 (3)
C16—C17—C18	121.7 (4)	C30—N4—Cu2	121.7 (3)
O3—C18—C13	125.0 (3)	C2—O1—C1	118.8 (3)
O3—C18—C17	118.8 (3)	C7—O2—Cu1	126.1 (2)
C13—C18—C17	116.2 (3)	C18—O3—Cu1	128.6 (2)
O4—C19—H19A	109.5	C17—O4—C19	119.2 (3)
O4—C19—H19B	109.5	Cu1—O5—H5A	117.4
H19A—C19—H19B	109.5	Cu1—O5—H5B	104.7
O4—C19—H19C	109.5	H5A—O5—H5B	108.6
H19A—C19—H19C	109.5	C21—O6—C20	118.5 (3)
H19B—C19—H19C	109.5	C26—O7—Cu2	128.2 (2)
O6—C20—H20A	109.5	C37—O8—Cu2	126.7 (2)
O6—C20—H20B	109.5	C36—O9—C38	118.1 (3)
H20A—C20—H20B	109.5	Cu2—O10—H10E	120.0
O6—C20—H20C	109.5	Cu2—O10—H10F	126.2
H20A—C20—H20C	109.5	H10E—O10—H10F	107.9
H20B—C20—H20C	109.5	O3—Cu1—O2	85.14 (12)
O6—C21—C22	124.3 (4)	O3—Cu1—N2	91.37 (14)
O6—C21—C26	113.7 (3)	O2—Cu1—N2	159.87 (18)
C22—C21—C26	121.9 (4)	O3—Cu1—N1	175.41 (12)
C21—C22—C23	120.5 (4)	O2—Cu1—N1	90.49 (12)
C21—C22—H22	119.8	N2—Cu1—N1	92.27 (15)
C23—C22—H22	119.8	O3—Cu1—O5	96.25 (12)
C24—C23—C22	119.4 (4)	O2—Cu1—O5	103.28 (13)
C24—C23—H23	120.3	N2—Cu1—O5	96.81 (18)
C22—C23—H23	120.3	N1—Cu1—O5	86.09 (14)
C23—C24—C25	122.0 (4)	O7—Cu2—O8	85.57 (11)
C23—C24—H24	119.0	O7—Cu2—N3	91.54 (13)
C25—C24—H24	119.0	O8—Cu2—N3	161.21 (17)
C24—C25—C26	120.1 (4)	O7—Cu2—N4	175.20 (13)
C24—C25—C27	118.3 (4)	O8—Cu2—N4	90.00 (11)
C26—C25—C27	121.6 (4)	N3—Cu2—N4	92.07 (15)
O7—C26—C25	125.1 (3)	O7—Cu2—O10	93.67 (12)
O7—C26—C21	118.8 (3)	O8—Cu2—O10	105.25 (13)
C25—C26—C21	116.0 (3)	N3—Cu2—O10	93.45 (17)
N3—C27—C25	127.9 (4)	N4—Cu2—O10	89.28 (15)

### Hydrogen-bond geometry (Å, °)

**Table:**

*D*—H···*A*	*D*—H	H···*A*	*D*···*A*	*D*—H···*A*
O10—H10E···O1	0.85	2.00	2.818 (4)	161
O10—H10F···O4	0.85	2.02	2.768 (4)	147
O5—H5A···O9^i^	0.85	2.05	2.779 (4)	143
O5—H5B···O6^i^	0.85	2.02	2.758 (4)	145

Symmetry codes: (i) *x*+1/2, −*y*+1/2, *z*.

## References

[bb1] Bruker (2001). *SAINT-Plus* Bruker AXS Inc., Madison, Wisconsin, USA.

[bb2] Bruker (2004). *APEX2* Bruker AXS Inc., Madison, Wisconsin, USA.

[bb3] Flack, H. D. (1983). *Acta Cryst.* A**39**, 876–881.

[bb4] Karlin, K. D. (1993). *Science*, **261**, 701–708. 10.1126/science.7688141 7688141

[bb5] Saha, P. K., Dutta, B., Jana, S., Bera, R., Saha, S., Okamoto, K. & Koner, S. (2007). *Polyhedron* **26**, 563–571.

[bb6] Shankar, R., Jain, A., Singh, A. P., Kociok-Kohn, G. & Molloy, K. C. (2009). *Inorg. Chem.* **48**, 3608–3616. 10.1021/ic802160a 19296601

[bb7] Sheldrick, G. M. (2003). *SADABS* University of Göttingen, Germany.

[bb8] Sheldrick, G. M. (2008). *Acta Cryst.* A**64**, 112–122. 10.1107/S0108767307043930 18156677

[bb9] Sui, Y., Hu, R.-H., Peng, J.-L. & Ng, S. W. (2007). *Acta Cryst.* E**63**, m2122.

[bb10] Ward, M. D. (2007). *Coord. Chem. Rev.* **251**, 1663–1677.

